# High risk-myelodysplastic syndrome following CAR T-cell therapy in a patient with relapsed diffuse large B cell lymphoma: A case report and literature review

**DOI:** 10.3389/fonc.2023.1036455

**Published:** 2023-01-20

**Authors:** Eugenia Accorsi Buttini, Mirko Farina, Luisa Lorenzi, Nicola Polverelli, Vera Radici, Enrico Morello, Federica Colnaghi, Camillo Almici, Emilio Ferrari, Andrea Bianchetti, Alessandro Leoni, Federica Re, Katia Bosio, Simona Bernardi, Michele Malagola, Alessandro Re, Domenico Russo

**Affiliations:** ^1^ Unit of Blood Diseases and Bone Marrow Transplantation, Cell Therapies and Hematology Research Program, Department of Clinical and Experimental Science, University of Brescia, ASST Spedali Civili di Brescia, Brescia, Italy; ^2^ Department of Molecular and Translational Medicine, Section of Pathology, University of Brescia, ASST Spedali Civili di Brescia, Brescia, Italy; ^3^ Stem Cell Laboratory, Section of Hematology and Blood Coagulation, Clinical Chemistry Laboratory, Diagnostics Department, ASST Spedali Civili di Brescia, Brescia, Italy; ^4^ Research Center Ail (CREA), Chair of Hematology Department of Clinical and Experimental Science, University of Brescia, ASST Spedali Civili di Brescia, Brescia, Italy; ^5^ Hematology Unit, ASST Spedali Civili di Brescia, Brescia, Italy

**Keywords:** CAR T-cell therapy, myelodysplastic syndrome, diffuse large B cell lymphoma, next generation sequencing, clonal hematopoiesis

## Abstract

**Background:**

Chimeric antigen receptor (CAR) T-cell therapy represents the most advanced immunotherapy against relapsed/refractory B cell malignancies. While cytokine release syndrome and immune effector cell-associated neurotoxicity syndrome are distinctive, known CAR T-cell acute adverse events, hematological toxicity has been increasingly reported. Cytopenia following CAR T-cell treatment is attributed in most cases to lymphodepletion regimens, bridging chemotherapy, or radiotherapy. However, when cytopenia becomes prolonged, the development of myelodysplastic syndrome (MDS) should be considered.

**Case presentation:**

We report a case of high risk (HR)-MDS following CAR T-cell therapy in a patient with relapsed diffuse large B cell lymphoma. Eight months after CAR T-cell infusion, the blood count showed progressive, worsening cytopenia and the bone marrow biopsy revealed multilineage dysplasia without excess of blasts associated with chromosome 7 deletion and *RUNX1* mutation. Next generation sequencing analysis, retrospectively performed on stored samples, showed a germ line *CSF3R* mutation, *CEBPA* clonal hematopoiesis, but no *RUNX1* lesion.

**Conclusion:**

We describe a case of HR-MDS, with deletion of chromosome 7 and acquisition of *RUNX1* mutation, developing after CAR T-cell therapy in a patient with clonal hematopoiesis (CH). Previous chemotherapy favored MDS onset; however, we could not exclude the fact that the impairment of immunosurveillance related to either lymphodepletion or CAR T-cell infusion may play a role in MDS development. Thus, we designed a multicenter prospective study (ClonHema-CAR-T-Study) to investigate if cytopenia after CAR T-cell treatment may be due to underling CH as well as the presence of secondary myeloid malignancies.

## Introduction

Chimeric antigen receptor (CAR) T-cell therapy has shown impressive efficacy in treating relapsed and refractory B-cell malignancies (1, 2). CAR T-cell therapy is frequently complicated by cytokine release syndrome (CRS) and immune effector cell-associated neurotoxicity syndrome (ICANS) (3). Besides these distinctive acute adverse events, hematological toxicity is emerging as the most relevant long term side effect (4). Cytopenias after CAR T therapy most likely have a multifactorial etiology due to active inflammation milieu, together with the impact of previous and lymphodepleting chemotherapy. Among the possible causes of persistent cytopenia, the development of secondary myeloid malignancies should be considered due to the previous history of chemo and/or radiation therapy and the significant impairment of immunosurveillance related to disease and CAR-T treatment (5). To the best of our knowledge, secondary myelodysplastic syndrome (MDS) clearly related to CAR T-cell treatment has not been described to date. We report the first case of high risk (HR)-MDS with chromosome 7 deletion and *RUNX1* mutation, which developed after CAR T-cell therapy in a patient with relapsed diffuse large B cell lymphoma (DLBCL).

## Case presentation

**Figure 1:**
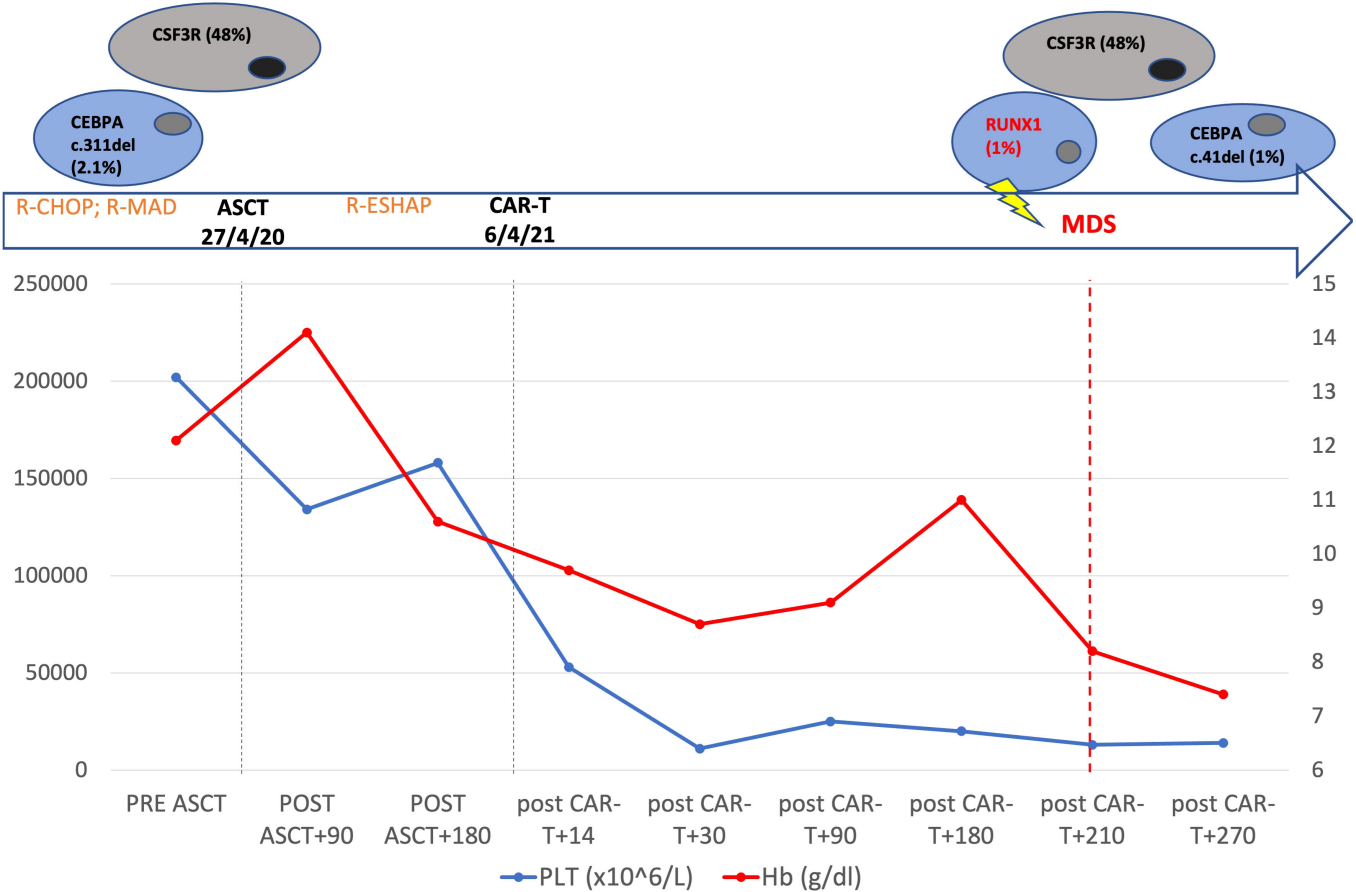
Patient’s timeline. Top: mutational profile detected by next generation sequencing. Middle: the main patient treatments. Changes of hemoglobin and platelets from the beginning of patient therapy to 9 months after CAR T cell-therapy are shown.

**Figure 2:**
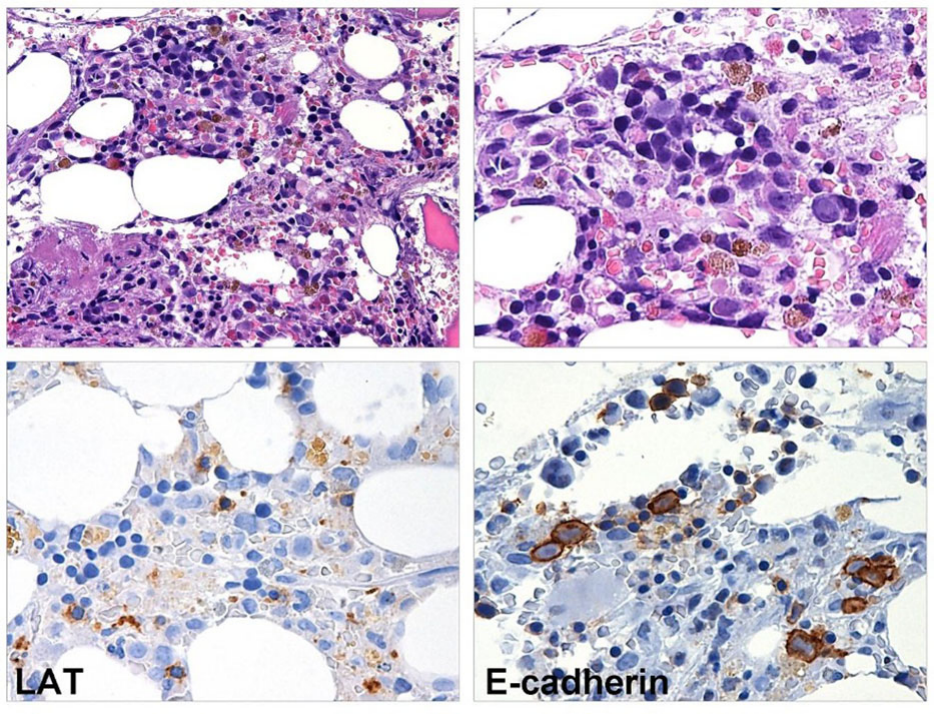
BM biopsy performed eight months after CAR T-cell infusion. Myeloid maturation is shifted to the left, granulocytes are rare and hyposegmented. The megakaryopoiesis is hypoplastic (LAT stain) and erythroid precursors show megaloblastoid features (E-caherin stain).

In August 2019, a 57-year-old woman with a previous history of diabetes mellitus and obesity was diagnosed with germinal-center DLBCL, Ann-Arbor stage IVs-A. At diagnosis, the patient presented cervical lymphadenopathies and spleen involvement. A bone marrow (BM) biopsy revealed the presence of 25% infiltrate of lymphocytes in the context of normal hematopoiesis. She was classified with age-adjusted International Prognostic Index (IPI) (score 3) and central nervous system (CNS-IPI) (score 4) high risk score. The patient was treated with four cycles of rituximab, cyclophosphamide, adriamycin, vincristine, and prednisolone (R-CHOP), achieving a computed tomography complete response (CR). Subsequently, she received one course of consolidation chemotherapy with rituximab, mitoxantrone, cytarabine, and dexamethasone (R-MAD), and one course of high dose cytarabine with stem cell collection showing a metabolic CR according to the Lugano criteria. In April 2020, autologous stem cell transplantation (ASCT) was performed using FEAM (fotemustine, etoposide, cytarabine, and melphalan) as a conditioning regimen. Unfortunately, in December 2020 the patient relapsed with enlarged retroperitoneal lymph-nodes, a hypodense liver, and uterine lesions. A BM biopsy was performed, and no signs of lymphoma infiltration or dysplasia were found. She was considered eligible for CAR T-cell treatment and underwent lymphocytes apheresis followed by two bridging courses of R-ESHAP (etoposide, cisplatin, cytarabine, and methylprednisolone). On the first day of standard lymphodepletion, the blood count showed grade II anemia, leukopenia, and grade I thrombocytopenia according to the Common Terminology Criteria of Adverse Events. The patient did not develop either CRS or ICANS, only an episode of fever occurred caused by *Staphilococcus haemolyticus*. Two months after CAR T-cell treatment, the patient was in CR and a BM biopsy presented normal cellularity, without dysplastic features or cytogenetic alterations. In the following months, the blood count showed a progressive normalization of leukocytes values, with hemoglobin ranging between 8.5 and 10.4 g/dl and platelets between 20 and 40 x10 (6)/L (**Figure 1**). Seven months later, the cytopenia worsened (grade III anemia, grade IV thrombocytopenia, grade III leukopenia), requiring recombinant human G-CSF and erythrocyte and platelet support. In December 2022, a BM biopsy ruled out lymphoma infiltration but revealed multilineage dysplasia without excess of blasts (**Figure 2**), associated with chromosome 7 deletion. A diagnosis of HR revised-IPSS MDS was made, and the patient was treated with azacitidine followed by allogeneic hematopoietic stem cell transplantation (allo-HCT).

## Discussion

Uni- or multi-lineage cytopenias are increasingly recognized as a side effect associated with CAR T-cell therapy. In the ZUMA trial, 17% of the patients treated with axicabtagene ciloleucel (axi-cel) presented with grade 3 or higher cytopenia at 3 month or later. Four patients developed MDS after CART-cell infusion, which was considered to be related to previous treatment (1). In the JULIET study, grade 3/4 thrombocytopenia persisted in 38% of patients 3 months after CAR T-cell treatment, while no case of grade 3/4 neutropenia was reported (2). Recently, Cordeiro et al. reported late adverse events after CAR T-cell treatment in patients who survived at least one year after therapy. Three of the 19 patients with ongoing CR had prolonged cytopenia without evidence of MDS after receiving CAR-T cells (5). Subsequent MDS occurred in four patients, and notably, two of these had cytogenetics abnormalities prior to CAR-T cell therapy. Apart from clinical trials, a multicenter analysis on 258 patients receiving axi-cel and tisa-cel showed prolonged neutropenia in 64% of patients (6). In addition, a case of BM aplasia after axi-cel treatment for DLBCL was submitted to allo-HCT (7) and a case of sustained myelosuppression after BCMA-CART therapy for relapsed myeloma was successfully treated with backup of autologous stem cells (8). While early cytopenia after CAR T infusion seems to be related to the myelotoxic effect of lymphodepletion, the development of late cytopenia likely has a multifactorial origin and it remains poorly understood (9). Some authors have shown that baseline cytopenia and elevated baseline levels of C-reactive protein (CRP) and ferritin were correlated with the duration of neutropenia after CAR T-cell therapy (6). Thus, the low marrow stem cell reserve, resulting from previous chemotherapy and the high levels of inflammation, were most likely correlated to hematologic toxicity. Based on this result, a CAR-HEMATOTOX score, capable to identify patients who are at high risk of developing significant cytopenia, was developed. In our patient, the CAR HEMATOTOX score was low, determined only by a high level of ferritin (1,016 ng/mL). Therefore, in order to explain the worsening of cytopenia 7 months after CAR T-cell treatment, we performed a BM biopsy and a diagnosis of HR-MDS was made. Notably, she developed a deletion of chromosome 7, which is considered one of the hallmark features of therapy related MDS (t-MDS). Secondary MDS following ASCT has an incidence estimated between 5 and 20%, and two main types of t-MDS have been described. The first is associated with previous exposure to topoisomerase II inhibitors, usually occurs 2–3 years after therapy, and translocations involving 11q23 or 21q22 are frequently associated. The second is related to alkylating agents, usually occurs 5–7 years after exposure, and loss or deletion of chromosomes 5 or 7 are the most common cytogenetic abnormalities described (10). Our patient was treated with both alkylating agents and topoisomerase II inhibitors before undergoing CAR T-cell therapy; therefore, she was at increased risk to developing MDS related to the mutagenic effect of previous chemotherapy. At the same time, we cannot exclude the fact that the CAR T-cell treatment may play a role in the development of the MDS. Recently, a case of diagnosis of acute myeloid leukemia (AML), which developed two months after CAR T-cell treatment for DLBCL was described by Falini at al (11). On top of AML diagnosis, next generation sequencing (NGS) detected mutations of the following genes: *DNMT3A* V626Gfs Ter4 (VAF: 46.2%), *RUNX1* splicing-site mutation (VAF: 16.8%), missense mutation N136K (VAF: 9.2%), and *PPM1D* S453 (VAF: 1.4%). Targeted sequencing of stored DNA from a BM sample taken before CAR T-cell treatment was also performed, which already showed the *DNMT3A* and the *PPM1D* mutations, but not *RUNX1*. Therefore, the patient had clonal hematopoiesis (CH) driven by *DNMT3A*, already present at the time of the DLBCL diagnosis, and the acquisition of *RUNX1* mutations 2 months after CAR T-cell infusion likely promoted the evolution to AML, most likely through cooperation with the deletion of chromosome 7.

In our case, NGS performed on a medullary sample using the commercial Myeloid Solution produced by SOPHiA GENETICS (SOPHiA GENETICS, Saint-Sulpice, Switzerland) 8 months after CAR T-cell treatment identified mutations of the following genes: *CSF3R* c.1319G>A; p.R440Q (VAF: 48%), *CEBPA* c.41del; p.P14R (VAF: 1%), and *RUNX1* c.508-2T>C (VAF: 1%). When we retrospectively performed the NGS analysis on a cryopreserved sample collected during the harvest of peripheral blood stem cells before ASCT, *CSF3R* c.1319G>A;p.R440Q (VAF: 48%) and *CEBPA* c.311_313del;p.G104del (VAF: 2.1%) were detected. *CSF3R* gene lesions are uncommon in MDS, occurring at a rate of 3%, and they are an age-independent and mostly IPSS-R independent risk factor for leukemia-free survival (12). In the setting of AML, *CSF3R* mutations are frequently associated with abnormalities of *RUNX1*, *CBFB*, *CEBPA*, and *NPM1 (* 13 *).* Conversely, *RUNX1* mutations occur in 8–23% of MDS, most commonly in the setting of therapy-related MDS, and are frequently detected in patients who develop AML, with a negative impact on survival (14).

In our case, the patient had a dominant clone with *CSF3R* mutation and a subclone with a *CEBPA* lesion before being submitted to ASCT. Considering the consistent variant allele fraction, without fluctuations over time, *CSF3R* mutation is likely to be of germ line origin, while we can consider the *CEBPA* lesion belonging to CH. Eight months after tisa-cel infusion, the cytopenia worsened dramatically and BM assessment revealed monosomy of 7 and the onset of *RUNX1* mutation (**Figure 1**). Thus, the patient most likely presented with a germ line *CSF3R* mutation and *CEBPA* CH before ASCT. Subsequently, the acquisition of chromosome 7 deletion and *RUNX1* mutation promoted the development of MDS. Currently, the patient has been submitted to allo-HCT from a haploidentical donor, and she is in CR after two months.

## Conclusion

This is the first case report of HR-MDS developing after CAR T-cell treatment and, in addition to the case described by Falini et al, the second in which deletion of chromosome 7 and acquisition of *RUNX1* are reported. The coincidental recurrence of these genomic alterations could be occasional, and this would certainly appear to be a limitation of our case report; however they may reveal a peculiar stepwise leukemogenic evolution starting from the presence of a pre-CAR T-cell CH trait.

The role of genotoxic damage related to previous chemotherapy and ASCT in patients with DLCBL is well-known. Conversely, the impairment of immunosurveillance, related either to the lymphoma or to T/B-cells aplasia (15) induced by lymphodepletion and CAR T-cell therapy, remain to be clarified and new studies are needed to investigate the impact of CAR T-cell therapy on risk of secondary hematological malignancies. The observation of these two first cases strongly suggest that a NGS genomic profile study for investigating the presence of CH before CAR-T-cell therapy should be performed. For these reasons, we have designed an Italian multicentric study, the ClonHema-CAR-T study, with the aim to evaluate CH before CAR T and monitor its clonal evolution after CAR T-cell infusion in the case of persistent cytopenia.

## Data availability statement

The original contributions presented in the study are included in the article/supplementary materials. Further inquiries can be directed to the corresponding author.

## Ethics statement

The studies involving human participants were reviewed and approved by Comitato etico di Brescia. The patients/participants provided their written informed consent to participate in this study. Written informed consent was obtained from the individual(s) for the publication of any potentially identifiable images or data included in this article.

## Author contributions

Manuscript writing: EAB. Review and editing: MF, LL, NP, VR, AT, EM, FC, CA, EF, AB, AL, FR, KB, SB, MM, AR, and DR. Figures: LL and MF. All authors contributed to the article and approved the submitted version.
